# The Chinese herbal formula Huoxiang Zhengqi for diarrhea-predominant irritable bowel syndrome (CHAIRS): a study protocol for a double-blinded randomized controlled trial

**DOI:** 10.1186/s13063-021-05444-w

**Published:** 2021-07-26

**Authors:** Xiaohui Guo, Meiling Xuan, Huan Zheng, Shumin Qin, Haomeng Wu, Shaogang Huang, Zehuai Wen

**Affiliations:** 1 Key Unit of Methodology in Clinical Research, Guangdong Provincial Hospital of Chinese Medicine, Guangzhou, China; 2 Guangdong Provincial Key Laboratory of Clinical Research on Traditional Chinese Medicine Syndrome, Guangzhou, China; 3 State Key Laboratory of Chinese Medicine Dampness Syndrome, Second Affiliated Hospital of Guangzhou University of Chinese Medicine, Guangzhou, China; 4 Department of Gastroenterology, Guangdong Provincial Hospital of Chinese Medicine, Guangzhou, China

**Keywords:** Traditional Chinese medicine, Chinese herbal formula, Huoxiang Zhengqi, Diarrhea-predominant irritable bowel syndrome, Study protocol

## Abstract

**Background:**

Diarrhea-predominant irritable bowel syndrome (IBS-D) is a common chronic digestive disease. Recent observational studies have reported that the Chinese herbal formula Huoxiang Zhengqi (HXZQ) can relieve IBS-D symptoms, but no high-level evidence is presented. Therefore, we want to evaluate the efficacy and safety of HXZQ for IBS-D patients.

**Methods:**

This is a double-blind, randomized, placebo-controlled trial. The 212 eligible patients with IBS-D will be randomly assigned to receive either HXZQ oral liquid or a placebo, at a 1:1 ratio, for 4 weeks with a 4-week follow-up period. Adequate relief will be the primary outcome measures. IBS symptom severity score, IBS quality-of-life questionnaire, EQ-5D-5L, and Chinese medicine symptom questionnaire will be the secondary outcome measures.

**Discussion:**

This trial aims to demonstrate the efficacy and safety of HXZQ for IBS-D, which is expected to be an effective IBS-D treatment.

**Trial registration:**

The trial was registered with the Chinese Clinical Trial Registry, ChiCTR1900026837. Registered on 24 October 2019.

**Supplementary Information:**

The online version contains supplementary material available at 10.1186/s13063-021-05444-w.

## Background

Irritable bowel syndrome (IBS) is a common functional gastrointestinal disorder characterized by recurrent abdominal pain associated with, or accompanied by, changes in bowel habits [1]. The current global prevalence of IBS is 11.2% [2], ranging from 4.7 to 25% in western countries and from 3.7 to 19.1% in eastern countries [3]. The prevalence in Asia is estimated to be between 6.5% and 10.1% [4] and between 4.6% - 5.6% in China [5]. There are several subtypes of IBS, with the diarrhea-predominant IBS (IBS-D) being the most common [6].

IBS’s pathogenesis is complex, and it is difficult to cure [7]. Countries around the world are increasingly concerned about IBS, so a lot of relevant studies have been carried out, hoping to provide evidence for the treatment of IBS. At present, there are no radical therapies for IBS. A number of different therapeutic interventions are available for the management of patients with IBS, including approved drugs and other interventions (e.g., dietary modifications, psychological interventions). Pharmacological interventions for the management of IBS-D include the US Food and Drug Administration-approved agents eluxadoline, rifaximin, and alosetron, as well as loperamide, smooth muscle antispasmodics, bile acid sequestrants, and antidepressants (i.e., tricyclic antidepressants, selective serotonin reuptake inhibitors) [8]. Chinese medicine (CM) has been used to treat functional bowel disorders for hundreds of years in China. A randomized placebo-controlled trial published in 1998 concluded that Chinese herbal formulations mitigate symptoms for some IBS patients [9]. A systematic review demonstrated that treating IBS with integrated traditional Chinese and western medicine was more effective than conventional western medicine alone. Thus, CM could be an attractive option, used in conjunction with conventional medicine, for managing IBS or its subtypes [10].

The Chinese herbal formula Huoxiang Zhengqi (HXZQ) is a classic prescription in CM practices. It is documented in the Prescriptions People’s Welfare Pharmacy (Taiping Huimin Hejiju Fang) published in the Song dynasty (early 12th century). HXZQ is composed of *Perilla frutescens* (L.) Britton (Zisu), *Atractylodes macrocephala* Koidz. (Baizhu), *Platycodon grandiflorum* (Jacq.) A.DC. (Jiegeng), *Pogostemon cablin* (Blanco) Benth. (Huoxiang), *Glycyrrhiza uralensis Fisch.* (Gancao), *Citrus reticulata* Blanco (Chenpi), *Magnolia officinalis Rehd*. et Wils. (Houpu), *Angelica dahurica* (Fisch.ex Hoffm.) Benth.et Hook.f. (Baizhi), *Poria cocos* (Schw.) Wolf. (Fuling), *Areca catechu* L. (Dafupi), and *Pinellia ternata* (Thunb.) Breit. (Banxia). Some of these Chinese herbs overlap with that from Bensoussan et al.’s [9] and Li et al.’s study reports [10]. Coming in various dosage forms of HXZQ such as capsules, granules, and oral liquid, it is often used to treat diseases related to the dampness pattern (Shi Zheng) in CM, such as gastrointestinal disorders and acute gastroenteritis [11]. Moreover, the results from an animal study and a clinical trial may provide an explanation for the rationale of the HXZQ intervention for IBS-D. Studies by Lu and Fang have shown that HXZQ has pharmacological effects such as spasmolysis, analgesia, bacteriostasis, regulating gastrointestinal motility, enhancing intestinal mucosal protection, and improving water, electrolyte, and metabolic disorders [12, 13].

Two randomized controlled trials, published in 2011 and 2003, respectively, have also shown that the modified HXZQ formula is an effective treatment for IBS-D [14, 15]. These two trials, however, were of low methodological quality and were thus unable to contribute to the evidence gap of HXZQ for IBS-D. Therefore, we conducted a double-blind randomized controlled trial to investigate the efficacy and safety of HXZQ for IBS-D.

## Methods/design

### Study design and setting

**Fig. 1:**
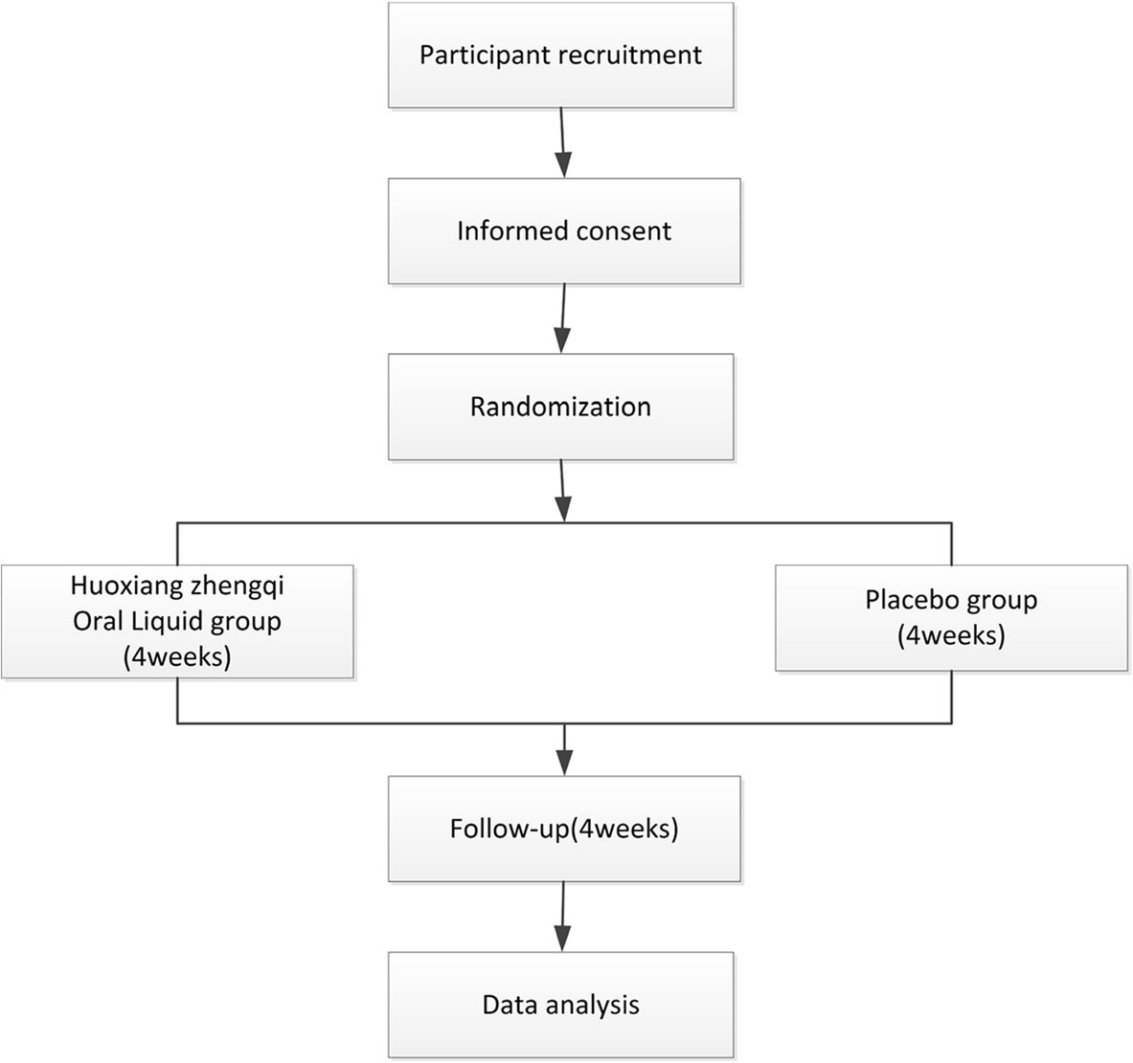
Flow chart of this study

This study is a multi-center, double-blind, randomized placebo-controlled trial. A flowchart of the trial is shown in Fig. 1. This trial will be conducted at 11 first-class hospitals in China in accordance with the principles of good clinical practice and the Declaration of Helsinki. The study protocol was approved by the ethics committees of the participating hospitals. The trial was registered with the Chinese Clinical Trial Registry (ID: ChiCTR1900026837). The protocol reporting follows the Standard Protocol Items for Clinical Trials 2013 (SPIRIT 2013) [16].

### Patients

All participants will be recruited from the following 11 research centers: Guangdong Provincial Hospital of Chinese Medicine, Chongqing Traditional Chinese Medicine Hospital, Peking University Third Hospital, Beijing Friendship Hospital of Capital Medical University, Shaanxi University of Traditional Chinese Medicine, Shuguang Hospital of Shanghai University of TCM, Chongqing Red Cross Hospital, Shenzhen Second People’s Hospital, Yuncheng Center Hospital, Xiangyang 1st People’s Hospital, and Hebei Provincial Hospital of Traditional Chinese Medicine.

Participants will be recruited through social media and recruiting advertisements in hospitals. After patients are screened for eligibility with the following criteria and sign informed consent, they will be enrolled in the study. During screening, enrolling, and withdrawing from the trial, all personal patient information will be kept strictly confidential within the scope of the law, except for the inspection or monitoring of the source data.

### Criteria for CM dampness pattern diagnosis

The diagnosis of CM dampness pattern refers to the Standard of Diagnosis and Curative Effect of Chinese Medicine Syndrome and Diseases [17] and the textbook of CM colleges and universities, the Diagnostics of Chinese Medicine [18]. Diagnosis of CM dampness pattern will be made by senior CM professional practitioner according to the characteristics of the disease based on the following criteria.

1. Common characteristics of CM dampness pattern
  Primary symptoms: mental fatigue, anorexia, loose stool or watery diarrhea, white sticky greasy tongue coating
  Secondary symptoms: heavy sensation in limbs, abdominal distension, pale tongue or pale teeth-marked tongue, soggy or moderate pulse
2. CM dampness pattern characteristics: loose stool or watery diarrhea, abdominal pain, fatigue, cold attack or aggravation, white sticky greasy tongue coating, weak pulse

Patients with two primary symptoms and one secondary symptom are considered to have the CM dampness pattern, as well as patients with one primary symptom plus two disease characteristics.

### Inclusion criteria

Patients who meet all of the following conditions will be included.

1. Those who met the diagnostic criteria for IBS-D according to Rome IV [1]
2. Those who were diagnosed as having a CM dampness pattern
3. Aged 18 to 70 years
4. Initial Irritable Bowel Syndromes Symptom Severity (IBS-SSS) score > 75
5. Patients had to be literate

### Exclusion criteria

Patients with one of the following conditions will be excluded.

1. Patients with serious diseases involving the heart, liver, kidneys, hematopoietic system or tumors, or patients with a history of serious nervous system or mental illness
2. Patients with warning signs (weight loss within 3 months of emaciation > 10%), hematochezia that is confirmed not to have been caused by hemorrhoids or anal fissure, diarrhea at night, fever, family history of colorectal cancer (or polyposis syndrome), inflammatory bowel disease (IBD), or celiac disease
3. Organic diseases of the digestive system or systemic diseases that affect digestive tract dynamics
4. Patients with a history of abdominal surgery (except cesarean section)
5. Those who are unwilling or unable to stop using drugs that affect the evaluation of interventions during the study
6. Pregnant and nursing women or women planning to become pregnant within 3 months
7. Those who have participated in other clinical trials within the past 3 months
8. Standard points on the Self-rating Anxiety Scale (SAS) > 50; standard points on the Self-rating Depression Scale (SDS) > 53
9. Glutamic pyruvic transaminase (ALT) or glutamic oxaloacetic transaminase (AST) double the upper limit of the normal range; total bilirubin (TBIL) or blood urea nitrogen (BUN) is 1.5 times higher the upper limit of the normal range
10. Patients with a history of allergy to drugs used in the study
11. Researchers believe that patients are not suitable for the study

### Sample size calculation

The sample size was calculated based on the primary outcome of the proportion of responders of adequate relief (AR). Calculations were made with PASS 11.0 (NCSS, LLC, Kaysville, UT, USA). According to the results of similar clinical trials [9, 19], we assume that HXZQ’s AR response rate is 60% and that the placebo’s is 30%. Thus, we estimated that for 80% power to detect a superiority margin of 10% on the RA response rates at a given 2-tailed type I error of 0.05, 106 patients will be needed in each group, after allowing for a 15% dropout rate. A total of 212 patients will be randomly allocated to the two groups at an equal ratio.

### Randomization and blinding

A center-stratified block randomization sequence generated by using SAS 9.2 (SAS Institute Inc., Cary, USA) has been completed by the Institute of Basic Research in Clinical Medicine (IBRCM), China Academy of Chinese Medical Science. All eligible participants will be randomly assigned to either the HXZQ group or the placebo group at a ratio of 1:1 through an interactive web response system. Investigators will log into the system to acquire participants’ treatment allocation. The randomization results will be kept confidential and maintained by IBRCM.

All patients and researchers, including investigators, study assistants, outcome assessors, statisticians, and other staff members will know neither patients’ allocation nor their treatment. The placebo should be identical to HXZQ in appearance, taste, weight, labeling, and packaging, but contain no active ingredients. HXZQ oral liquid and the matching placebo will also be examined based on appearance, taste, and weight by the triangulation method.

### Interventions

In the experimental group, participants will receive 20 ml of HXZQ oral liquid twice a day for 4 weeks. Participants in the control group will receive 20 ml of a placebo oral liquid twice a day for 4 weeks. After the treatment, all participants will be followed up for 4 weeks. During the study period, patients will not be allowed to receive other IBS-D treatments.

The HXZQ and placebo oral liquids will be manufactured by Taiji Group Chongqing Fuling Pharmaceutical Co. Ltd. (Chongqing, China) according to the requirements of good manufacturing practice. The placebo will be made from glycyrrhizin, bitterant, caramel color, and trace amounts of cinnamon extract and ginger juice. It will meet the hygienic requirements of the oral liquid product.

### Drugs and therapy prohibited during the trial

Any medications which might treat IBS-D and drugs to relieve abdominal pain and diarrhea will be prohibited. Other prohibited drugs and therapies include any traditional Chinese medicine except for the study drugs, non-pharmaceutical therapy of CM, such as acupuncture, moxibustion, massage, or cupping for IBS-D.

During the trial, if patients develop intolerable abdominal pain or diarrhea, they can take rescue medications as directed by their doctors. The name and dosage of the rescue medication will be recorded in the patient's diary.

### Monitoring compliance

Participants will be requested to record the weekly number of HXZQ they consume in the patient diaries. Any unused HXZQ oral liquid must be returned at each visit. The quantity of consumed and returned HXZQ will then be recorded in the CRF to measure compliance. Patients with compliance rates equal to or greater than 80% will be considered as having high compliance.

### Outcome measurements

#### Primary outcome

The primary outcome is adequate relief (AR) responder rates of the treatment period. AR is used to evaluate the degree of IBS symptom alleviation. During treatment and follow-up, participants will be asked the following question every week: “Over the past 7 days, have you had adequate relief of your irritable bowel syndrome pain and discomfort?” Participants will answer either “yes” or “no.” A responder is defined as those who answer “yes” for at least 2 of the 4 weeks, and the rest will be considered non-responders [20–22]. We will analyze the AR responder rates for the treatment (weeks 1–4) and follow-up (weeks 5–8) periods separately.

#### Secondary outcomes

Secondary outcomes include the Irritable Bowel Syndromes Symptom Severity Score (IBS-SSS), the Irritable Bowel Syndrome-Quality of Life Questionnaire (IBS-QOL), EuroQol-5-Dimensions-5-Level (EQ-5D-5L), and a Chinese medicine symptoms questionnaire.

IBS-SSS is used to evaluate IBS severity [23]. The participants will complete the questionnaires during the run-in period or at baseline (week 0), the end of the treatment period (week 4), and the follow-up period (week 8). IBS-QOL is a quality of life scale for IBS patients with good measurement validity [24]. EQ-5D-5L is a standardized health status measure developed by the EuroQol Group in order to provide a simple, generic measure of health for clinical and economic appraisal [25]. The Chinese medicine symptoms questionnaire is used to record patients’ symptoms to observe any changes in them. All secondary outcomes measurements will be completed at baseline (week 0), the end of the treatment period (week 4), and the end of the follow-up period (week 8).

### Safety assessment

All patients will be asked about the occurrence of any unfavorable or unintended effects, or any adverse events (AEs). AEs will be recorded on a case report form (CRF) and addressed appropriately. Before and after the treatment (weeks 0 and 4), all participants will undergo electrocardiogram and laboratory testing including a routine blood test, kidney function test, liver function tests, urine and stool tests. Investigators will pay attention to any abnormal changes indicated by the above examination results. All AEs will be assessed and analyzed, and causality between the study drugs will be evaluated according to the WHO Uppsala Monitoring Center System for Standardized Case Causality Assessment [26]. An independent Data Safety and Monitoring Committee (DSMC) will also assess the safety data during the trial. Severe AEs or severe adverse reactions should also be reported to the DSMC, the hospital ethics committee, and the research team, within 24 h. The content and timing of the enrollment, intervention, and evaluation are shown in Table 1.

**Table 1:** Content for the schedule of enrollment, interventions and assessments

Items	Study phase	Screening	Baseline	Treatment period				Follow-up period			
	Time	-1W	0W	1W	2W	3W	4W	5W	6W	7W	8W
Subjects selection	Enrolment	√									
	Eligibility screen	√									
	Informed consent	√									
	Randomization		√								
Intervention	HXZQ group			√	√	√	√				
	Placebo group			√	√	√	√				
Outcome assessments	Baseline assessments		√								
	AR		√	√	√	√	√	√	√	√	√
	IBS-SSS, IBS-QOL, EQ-5D-5L, CM symptoms questionnaire		√				√				√
Safety assessment	Laboratory tests		√				√				
	Adverse events										

*CM* Chinese medicine, *IBS-D* Diarrhea-predominant irritable bowel syndrome, *HXZQ* Huoxiang Zhengqi oral liquid, *AR* Adequate relief, *IBS-SSS* Irritable Bowel Syndromes Symptom Severity Score, *IBS-QOL* Irritable Bowel Syndrome-Quality of Life Questionnaire, *EQ-5D-5L* EuroQol-5-Dimensions-5-Level

### Data management and quality control

Prior to the study, relevant personnel involved in data collection and management, including investigators, study assistants, nurses, and other staffs, will receive at least 4 h of training, in order to maintain protocol implementation compliance. The completed CRFs will be reviewed by the monitor, and then transferred to the data manager for data entry. An electronic data management system will be adopted for data entry and management. The system will be equipped with a series of logical consistency checks for data entry, and the data manager will also be able to check the data manually through the system. Investigators will return the feedback for the query to the data manager to ensure that the data can be uploaded to the data management system in a timely, accurate and complete manner. Trial monitoring will be conducted regularly with SOPs by the Beijing Yaohai Ningkang Pharmaceutical Technology Co. Ltd. (Beijing, China). Audits will be executed regularly by IBRCM at the China Academy of Chinese Medical Sciences.

### Statistical analysis

Data analysis will be conducted in accordance with a statistical analysis plan prepared in advance. Statistical analysis will adhere to the intent-to-treat (ITT) and per protocol (PP) principles, and be performed with either SPSS 18.0 (IBM SPSS Inc, Armonk, New York, USA) or SAS 9.2 (SAS Institute Inc., Cary, USA) by independent statisticians at IBRCM. Statistical analyses will be conducted with a two-sided significance level of 0.05.

Patients’ baseline characteristics will be reported with descriptive statistics. Baseline data will be presented using frequencies, percentages, mean, standard deviation (SD), median and interquartile range according to the scales of the measurement and distributions. For primary outcome, AR responder rate, the data analysis will be performed on the ITT set, defined as all patients randomized to study treatment. AR responder rate will be compared between both groups at 4 weeks and 8 weeks after treatment considering superiority comparison between two groups by 95% confidence interval (CI) method. A chi-square test will be used for categorical data, and a t-test will be used for continuous data if the data distribution is appropriate. Missing data caused by withdrawal, loss to follow-up, or dropout will be addressed with multiple imputation methods. Sex, age, course of disease, and center effect will be considered covariates in a logistic regression or general linear model. If necessary, various sensitivity analyses will be conducted to assess the unimputation methods and complete case analyses. A safety analysis set (SS) will be used for safety evaluation and analysis. For safety analysis, the two groups’ incidence of adverse reactions will be compared by using a chi-square test or Fisher’s exact test. The AEs’ severity and the causality between AEs and the study drug should also be considered. If there is a significant amount of adverse reactions, the relationship with medication duration and baseline characteristics should be analyzed.

## Discussion

This study is a double-blind, randomized, placebo-controlled trial on HXZQ—a proprietary Chinese formula for IBS-D. This trial will be conducted at 11 hospitals, each in different Chinese cities. The trial will assess whether HXZQ benefits IBS-D patients.

The results from Lu’s and Fang’s studies that HXZQ has pharmacological effects such as spasmolysis, analgesia, regulating gastrointestinal motility, and enhancing intestinal mucosal protection [12, 13] have presented some explanation for the rationale of the HXZQ for IBS-D. While two RCTs [14, 15] showed the effect of modified HXZQ formulas for IBS, the methodological quality was low. So this trial is expected to contribute to high-quality evidence gap for IBS-D treatment with HXZQ.

Although AR is a patient-reported global assessment outcome, it represents whether the therapeutic effect has clinical significance. This reflects the goal of the drug therapy [27]. AR responder rates were determined over the first 4 weeks of treatment (weeks 1–4) and the second 4 weeks of follow-up (weeks 5–8) based on the patient’s diary. Although AR may have a variation as subjective patient-reported data, double-blinding will minimize the expectation bias from patients and investigators during the trial.

There will be several challenges in implementing this trial. Considering the difficulty of concocting a matched placebo, we have repeatedly adjusted the placebo ingredients to render its taste similar to that of HXZQ, to the greatest extent possible. The matched placebo and the HXZQ oral liquid have also been examined by the triangulation method in that HXZQ and the matched placebo will be evaluated blindly by more than a dozen nurses, patients and external personnel to ensure that patients cannot distinguish the two. AR at the end of the treatment period is a primary outcome in this study. If a patient fails to report on time, it may cause recall bias or missing data. Therefore, we will ask the investigators and study assistants to call the patients to ask the above questions every week, and record patients’ responses, reminding the patients to complete their responses on time. We believe can collect adequate data for AR assessment at least in the first 4 weeks of treatment. Patients with insufficient diary data for AR response will be categorized as nonresponders. In addition, the trial only evaluated HXZQ treating patients with IBS-D for 4 weeks and did not carry on the long-term effect and the recurrence assessment. However, according to relevant literature [28], the shortest effective treatment period can be expected to be 4 weeks, with effectiveness assessed throughout the treatment period.

We hope the results of this trial provide high-quality evidence on the efficacy and safety of HXZQ oral liquid versus placebo for IBS-D. At present, the clinical treatment of IBS-D still lacks an effective treatment plan. This study may provide a viable drug treatment option for IBS-D patients, especially for those with CM dampness patterns.

## Trial status

The protocol version is V1.1/20190826. The recruitment started in February 2020 and is meant to last until December 2021.

## Supplementary Information

**Additional file 1.** SPIRIT 2013 Checklist: Recommended items to address in a clinical trial protocol and related documents.

## Data Availability

Data sharing is not applicable to this article as no databases were generated or analyzed during the current stage. However, data are available from the corresponding author upon reasonable request after the study is complete.

## Abbreviations

IBS-D: Diarrhea-predominant irritable bowel syndrome
HXZQ: Huoxiang Zhengqi oral liquid
AR: Adequate relief
IBS-SSS: Irritable Bowel Syndromes Symptom Severity Score
IBS-QOL: Irritable Bowel Syndrome-Quality of Life Questionnaire
EQ-5D-5L: EuroQol-5-Dimensions-5-Level
